# High-throughput miRNA profiling of human melanoma blood samples

**DOI:** 10.1186/1471-2407-10-262

**Published:** 2010-06-07

**Authors:** Petra Leidinger, Andreas Keller, Anne Borries, Jörg Reichrath, Knuth Rass, Sven U Jager, Hans-Peter Lenhof, Eckart Meese

**Affiliations:** 1 Institute of Human Genetics, Medical School, Saarland University, Building 60, 66421 Homburg/Saar, Germany; 2 Febit biomed gmbh, Im Neuenheimer Feld 519, 69120 Heidelberg, Germany; 3 Biomarker Discovery Center Heidelberg, 69120 Heidelberg, Germany; 4 Clinic for Dermatology, Venerology and Allergology, Medical School, Saarland University, 66421 Homburg/Saar, Germany; 5 Praxis für Dermatologie, 66280 Sulzbach/Saar, Germany; 6 Center for Bioinformatics, Saarland University, Building E 1 1, 66041Saarbruecken, Germany

## Abstract

**Background:**

MicroRNA (miRNA) signatures are not only found in cancer tissue but also in blood of cancer patients. Specifically, miRNA detection in blood offers the prospect of a non-invasive analysis tool.

**Methods:**

Using a microarray based approach we screened almost 900 human miRNAs to detect miRNAs that are deregulated in their expression in blood cells of melanoma patients. We analyzed 55 blood samples, including 20 samples of healthy individuals, 24 samples of melanoma patients as test set, and 11 samples of melanoma patients as independent validation set.

**Results:**

A hypothesis test based approch detected 51 differentially regulated miRNAs, including 21 miRNAs that were downregulated in blood cells of melanoma patients and 30 miRNAs that were upregulated in blood cells of melanoma patients as compared to blood cells of healthy controls. The tets set and the independent validation set of the melanoma samples showed a high correlation of fold changes (0.81). Applying hierarchical clustering and principal component analysis we found that blood samples of melanoma patients and healthy individuals can be well differentiated from each other based on miRNA expression analysis. Using a subset of 16 significant deregulated miRNAs, we were able to reach a classification accuracy of 97.4%, a specificity of 95% and a sensitivity of 98.9% by supervised analysis. MiRNA microarray data were validated by qRT-PCR.

**Conclusions:**

Our study provides strong evidence for miRNA expression signatures of blood cells as useful biomarkers for melanoma.

## Background

For many human cancer entities, there is still a lack of high-performing biomarkers. In the past years, different tumor markers have been identified not only in tissue but also in blood, urine, or saliva of cancer patients. Several types of biomarkers can be distinguished. Prognostic biomarkers differentiate between "good outcome" and "bad outcome" tumors. Predictive biomarkers assess the probability for a treatment response, and pharmacodynamic biomarkers can be used to guide dose selection for certain drugs [1]. Furthermore, early detection biomarkers can indicate the onset of a tumor. Most recently, microRNAs (miRNAs) have been introduced as new cancer markers in the biomarker landscape and are suggested as targets or future therapy approaches [2,3]. MiRNAs are endogenous small noncoding RNAs that regulate translation and transcription. The expression of miRNAs has been demonstrated to be highly specific for tissues and developmental stages. In addition, miRNAs appear to contribute to the molecular classification of tumors [4].

Recent proof-of-principle studies indicate that analysis of miRNA expression in sera and peripheral blood cells is a promising approach for a blood-based diagnosis of cancer and other diseases [5-9]. We recently showed that complex miRNA expression patterns, rather than single miRNAs, can serve as biomarker signatures. Specifically, we were able to separate patients with different human diseases, including lung cancer [10] and Multiple Sclerosis [11] from healthy individuals by blood testing.

In this study, we describe a highly specific miRNA expression profile for melanoma patients. Malignant melanomas represent the most aggressive form of skin cancer. According to the World Health Organization (WHO) the number of melanoma cases continues to increase in incidence, faster than any other type of cancer. Melanoma accounts for about 4% of skin cancer cases but for as many as 74% of all deaths of skin cancer. The 5-year survival rate is as low as 5% for patients with advanced melanoma [12].

Currently, there is no promising standard therapy available for the treatment of patients with melanoma in an advanced stage. In order to improve prognosis it is crucial to detect melanoma in a very early stage, especially with metastasis occuring very early in the progression of the disease.

Several studies described altered miRNA expression fingerprints in melanoma with the majority of these studies analyzing miRNA expression in formalin fixed paraffin embedded cancer tissue [13-15] and few studies analyzing cancer cell lines [16,17]. Most notably, miRNAs have also been shown to be significantly correlated with metastasis in melanoma [18].

As of now there is, however, no evidence for altered miRNA expression in peripheral blood samples of melanoma patients. Here we used the Geniom Real Time Analyzer (GRTA) microarray platform (febit biomed GmbH, Heidelberg) to analyze all human miRNAs as annotated in the Sanger miRBase version 12.0 [19-21].

In total, we analyzed 35 blood samples of melanoma patients and 20 blood samples of healthy individuals. The 35 melanoma samples include a test set of 24 samples and an independent validation set of 11 melanoma samples.

As analysis tools we employed different well known statistical measures, including t-test, Wilcoxon Mann-Whitney test (WMW), a linear model with p-values computed by an empirical bayes approach (limma) [22,23], Area under the receiver operator characteristic curve (AUC), and fold changes. We classified melanoma patients and healthy subjects using Support Vector Machines (SVM) [24] that have been evaluated with a filter subset selection technique and standard 10-fold cross validation (CV).

Our study provides evidence for a novel and complex miRNA expression profile in blood cells of melanoma patients.

## Methods

### Samples

The study was conducted in compliance with the Helsinki Declaration. The local ethics committee ("Ärztekammer des Saarlandes") approved the study. All participants of this study have given written informed consent. The 35 blood samples of melanoma patients were collected in two independent institutions. We used the 24 blood samples from one institution as test set and the 11 blood samples from the second institution as validation set. The control samples were obtained from 20 healthy volunteers. Information on age and sex of all blood donors and detailed clinical informations for all melanoma patients is given in the Additional Files (Additional File 1, Table S1).

### miRNA extraction and microarray screening

Blood drawing of melanoma patients and isolation of RNA was performed as previously described [10]. Samples were analyzed with the Geniom Realtime Analyzer (GRTA, febit gmbh, Heidelberg, Germany) using the Geniom Biochip miRNA homo sapiens. Each array contains 7 replicates of 866 miRNAs and miRNA star sequences as annotated in the Sanger miRBase 12.0 [19-21]. Sample labeling with biotine was carried out by microfluidic-based enzymatic on-chip labeling of miRNAs (MPEA [25]).

In brief, following hybridization of the miRNA with the Geniom biochip for 16 hours at 42°C the biochip was washed automatically and a program for signal enhancement was processed with the GRTA. The detection pictures were evaluated using the Geniom Wizard Software. For each array, the signal intensities for all miRNAs were extracted from the raw data file. As each miRNA is spotted in seven replicates, we obtained seven intensity values for each miRNA. Following background correction, we calculated the median of the seven replicate intensity values for each miRNA. We applied quantile normalization, to normalize the data across different arrays [26]. Further analysis was carried out using the normalized and background subtracted intensity values.

The microarray data were deposited in the publicly available database Gene Expression Omnibus (GEO; http://www.ncbi.nlm.nih.gov/projects/geo/, GSE20994).

### Measures for single biomarker analysis

First, we analyzed the miRNA expression to detect miRNAs that show a different expression in different groups of blood donors. To this end, we applied different statistical measures to monitor differences between these measures. The set of approaches contains parametric t-test (unpaired, two-tailed), Wilcoxon Mann-Whitney test (WMW, unpaired, two-tailed), a linear model with p-values computed by an empirical Bayes approach (limma) [22,23], the area under the receiver operator characteristics curve (AUC) and fold quotients. The AUC is here defined as the "value" of a miRNA with respect to its ability to separate two different groups of blood donors. We calculated the AUC for each miRNA as follows: the normalized intensities of all miRNAs for all blood samples from melanoma patients and healthy controls were used as threshold values. For all thresholds *t*, we considered RNA from blood of melanoma patients that generate miRNA intensity values above *t *as true positives (TP), RNA from blood of melanoma patients that generate miRNA intensity values below *t *as false negatives (FN), RNA from blood of healthy subjects that generate miRNA intensity values below *t *as true negatives (TN), and RNA from blood of healthy subjects that generate miRNA intensity values above *t *as false positives (FP). Likewise for all thresholds, specificity (TN/(TN+FP)) and sensitivity (TP/(TP+FN)) were computed. The Receiver Operator Characteristics (ROC) curve shows the sensitivity as function of one minus the specificity. AUC values can range from 0.5 to 1. An AUC of 0.5 for a miRNA means that the distribution of intensity values generated by RNA from blood of melanoma patients and healthy subjects cannot be distinguished. The more the AUC value of a miRNA differs from 0.5, the better this miRNA is suited to separate between the two groups of blood donors (melanoma patients and healthy individuals). An AUC of 1 corresponds to a perfect separation.

For all hypothesis tests, the resulting p-values were adjusted for multiple testing by Benjamini-Hochberg [27,28] adjustment. We compared detected sets of relevant miRNAs by using venn-diagrams.

### Cluster Analysis and Principal Component Analysis

We carried out a hierarchical clustering approach to detect clusters of miRNAs and blood samples. In detail, we applied bottom up complete linkage clustering and used the Euclidian distance measure.

In addition, we carried out a standard principal component analysis (PCA) and provide scatter plots of the first versus second principal component [29,30].

### Classification analysis

In addition to the single biomarker analysis and unsupervised clustering we also carried out classification of samples using miRNA expression patterns by applying Support Vector Machines (SVM, [24]) as implemented in the R e1071 package [31]. In detail, different kernel (linear, polynomial, sigmoid, radial basis function) SVM have been evaluated, with the cost parameter sampled from 0.01 to 10 in decimal powers. The measured miRNA expression profiles were classified using 100 repetitions of standard 10-fold cross validation (CV). As a subset selection technique we applied a filter approach based on t-test. In detail, the *s *miRNAs with lowest p-values were computed on the training set in each fold of the CV, where *s *was sampled from 1 to 866 (corresponding to 866 analyzed human miRNAs and miRNA star sequences). The respective subset was used to train the SVM and to carry out the prediction of the test samples. As result, the mean accuracy, specificity, and sensitivity were calculated together with the 95% Confidence Intervals (95% CI) for each subset size. To check for overtraining we applied permutation tests. Here we sampled the class labels (melanoma and healthy) randomly and carried out classifications using the permuted class labels. All statistical analysis was performed using R [31].

### quantitative Real Time-PCR

To validate the microarray results we performed quantitative Real Time-PCR (qRT-PCR). We analyzed 13 miRNAs that showed significant deregulation in the microarray experiments, including hsa-miR-106b, hsa-miR-107, hsa-miR-1280, hsa-miR-151-3p, hsa-miR-17*, hsa-miR-18a, hsa-miR-199a-5p, hsa-miR-20a, hsa-miR-20b, hsa-miR-30a, hsa-miR-362-3p, hsa-miR-550*, and hsa-miR-664, using TaqMan^® ^MicroRNA Assays (Applied Biosystems). The qRT-PCR was performed on ten melanoma samples and ten samples of healthy individuals. We used RNU48 as endogenous control.

## Results

Using the Geniom Realtime Analyzer microarray platform, we analyzed the expression of 866 miRNAs in blood cells of 20 healthy volunteers and 35 patients with melanoma. Out of these 35 patients, 31 (88.57%) had melanoma of clinical stages 0, IA, IB, IIA, or IIB. One patient (2.86%) had a stage IIIB melanoma, and three patients (8.57%) had stage IV melanoma (see Additional file 1, Table S1).

**Figure 1:**
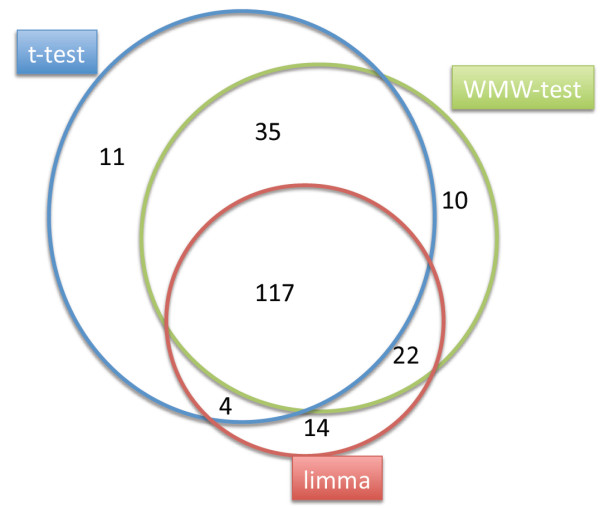
**Venn-diagram for the comparison of three hypothesis tests**. Numbers of miRNAs that are differentially expressed in blood cells of melanoma patients as compared to healthy controls. The three-way venn-diagram indicates the numbers of miRNA identified as significant by t-test (blue circle), Wilcoxon Mann-Whitney test (green circle), and a linear model with p-values computed by an empirical bayes approach (limma, red circle). The numbers inside the intersections of circles denotes the number of miRNAs significant for two or three of the tests.

To achieve improved statistical significance we compared the three hypothesis tests including t-test, Wilcoxon Mann-Whitney test (WMW), and a linear model with p-values computed by an empirical bayes approach (limma). We considered all miRNAs with adjusted p-value below 0.001 to be significant. All tests combined identified 213 miRNAs that showed a different expression in blood of melanoma patients as compared to the blood of healthy individuals. A more detailed view on the results of the three hypothesis tests is presented as three-way venn-diagram in Figure 1. In total, 117 miRNAs were detected in all three tests, additional 35 in both the WMW test and the t-test, additional 22 in both the empirical bayes test and the WMW test, and additional 4 in both the empirical bayes test and the t-test. With 167 miRNAs the t-test detected the highest number of deregulated miRNAs. Since the majority of the 213 miRNAs was identified in all three tests, the data demonstrate a high concordance of the employed hypothesis tests.

To further specify our search for miRNAs differentially expressed between blood of melanoma patients and of healthy controls, we added two additional filters. First, we only considered miRNAs that were at least 2-fold up- or down-regulated in their expression level in blood cells of melanoma patients compared to blood cells of healthy controls. Second, we calculated the median intensity values combined for all melanoma and combined for all normal blood samples and excluded miRNAs, with a combined median intensity value below 100 in either melanoma or in normal blood samples. Median intensity values lower than 100 are likely to be due to background noise. By using this high stringent threshold we tried to avoid false positives. Using these two empirically determined thresholds we identified 51 differentially expressed miRNAs, including 21 miRNAs downregulated and 30 miRNAs upregulated in blood cells of melanoma patients compared to blood cells of healthy individuals. Table 1 shows the 51 significantly deregulated miRNAs sorted by their AUC value. We validated the microarray results of 13 out of the above mentioned 51 miRNAs by using qRT-PCR. We analyzed ten randomly selected samples of melanoma patients and ten randomly selected samples of healthy controls that have already been analyzed by microarray. We found a correlation of 0.93 between the microarray data and the qRT-PCR data. Table 2 comprises the comparison of the fold changes in the miRNA expression between the analyzed melanoma and control samples of the microarray results and the qRT-PCR results.

**Table 1:** 51 significantly deregulated miRNAs sorted by their AUC value.

miRNA	median melanoma	median normal	fold change	wmw adjp	ttest adjp	limma adjp	AUC
hsa-miR-452*	189.7	633.3	0.3	0	0.00046	0	0.99
hsa-miR-216a	89.1	197.3	0.5	0.000001	1.7E-05	0.000039	0.98
hsa-miR-186	206.5	26.2	7.9	0.000001	0	0	0.97
hsa-let-7d*	178.6	37.7	4.7	0.000001	0	0	0.96
hsa-miR-17*	433.5	941.8	0.5	0.000001	0	0	0.96
hsa-miR-646	150.9	350.6	0.4	0.000001	0.00038	0	0.96
hsa-miR-217	86.3	183.8	0.5	0.000001	0.00055	0.000003	0.96
hsa-miR-621	178.6	486.7	0.4	0.000001	0.00011	0	0.95
hsa-miR-517*	109.9	230.8	0.5	0.000001	0.00022	0	0.95
hsa-miR-99a	217.3	85	2.6	0.000002	0	0	0.95
hsa-miR-664	557	173	3.2	0.000002	0	0	0.94
hsa-miR-593*	175.4	356.7	0.5	0.000002	0.00027	0	0.94
hsa-miR-18a*	397.4	135	2.9	0.000002	0	0	0.94
hsa-miR-145	358	94.6	3.8	0.000002	0	0	0.94
hsa-miR-1280	6779.6	2676.2	2.5	0.000002	0	0	0.93
hsa-let-7i*	122.8	281.4	0.4	0.000003	0.00045	0	0.93
hsa-miR-422a	279.2	104.5	2.7	0.000004	0	0	0.92
hsa-miR-330-3p	213.1	443.2	0.5	0.000004	0.00052	0	0.92
hsa-miR-767-5p	107.1	232.4	0.5	0.000004	0.00022	0.000001	0.92
hsa-miR-183*	195.9	87.7	2.2	0.000004	1E-06	0	0.92
hsa-miR-1249	144.8	46.1	3.1	0.000004	0	0.000004	0.92
hsa-miR-20b	2163.5	5665.8	0.4	0.000004	2E-06	0.000001	0.92
hsa-miR-509-3-5p	157	371.4	0.4	0.000004	0.00046	0	0.92
hsa-miR-519b-5p	72.5	155.1	0.5	0.000004	2.9E-05	0.000398	0.92
hsa-miR-362-3p	449	167.8	2.7	0.000004	4E-06	0	0.92
hsa-miR-501-5p	106.5	27.8	3.8	0.000004	0	0.000002	0.92
hsa-miR-378*	103.7	29.4	3.5	0.000004	0	0.000002	0.92
hsa-miR-365	160.5	65.1	2.5	0.000006	0	0.000001	0.91
hsa-miR-151-3p	999	422.6	2.4	0.000006	1E-06	0	0.91
hsa-miR-342-5p	196.8	92.1	2.1	0.000008	1E-06	0.000003	0.91
hsa-miR-328	175.4	32.3	5.4	0.000008	0	0.000001	0.9
hsa-miR-181a-2*	154.8	64.7	2.4	0.000016	4E-06	0.000004	0.89
hsa-miR-518e*	88.1	196.4	0.4	0.000019	0.00045	0.000586	0.89
hsa-miR-362-5p	245.4	119.5	2.1	0.000023	8E-06	0.000001	0.88
hsa-miR-584	198.2	46.9	4.2	0.000023	1.5E-05	0.000008	0.88
hsa-miR-550*	808.5	313.8	2.6	0.000024	2.6E-05	0.000003	0.88
hsa-miR-30a	682.9	334.8	2	0.000027	4E-06	0.000002	0.88
hsa-miR-221*	54.3	113.8	0.5	0.000029	0.00011	0.00039	0.88
hsa-miR-361-3p	263.9	99	2.7	0.000033	2E-06	0.000003	0.88
hsa-miR-625	185.8	63.3	2.9	0.000037	1.7E-05	0.000038	0.87
hsa-miR-146a	326.8	161.8	2	0.000037	3.9E-05	0.000003	0.87
hsa-miR-214	172.3	383.4	0.4	0.000042	0.00038	0.000001	0.87
hsa-miR-106b	8639.8	18881	0.5	0.000044	8.5E-05	0.000019	0.87
hsa-miR-18a	1060.8	2560	0.4	0.000053	0.00074	0.000013	0.86
hsa-miR-30e*	101.7	47.8	2.1	0.000022	5E-06	0.000098	0.86
hsa-miR-125a-5p	370.8	147.4	2.5	0.000059	3.3E-05	0.000001	0.86
hsa-miR-142-3p	105.3	2	53	0.000082	1.7E-05	0.000009	0.85
hsa-miR-107	725.8	1938.9	0.4	0.000092	0.00097	0.000034	0.85
hsa-miR-20a	3254.3	7282.8	0.4	0.000134	0.00016	0.000062	0.84
hsa-miR-22*	117.7	45	2.6	0.000193	3.7E-05	0.000138	0.83
hsa-miR-199a-5p	551.4	267.9	2.1	0.000201	0.00066	0.000042	0.83

adjp = adjusted p-value, AUC = area under the receiver operator charcteristic curve, wmw = Wilcoxon Mann-Whitney test, limma= linear model with p-values computed by an empirical bayes approach

**Table 2:** Comparison of the miRNA expression fold changes between the microarray and qRT-PCR results.

miRNA	fold change qRT-PCR	fold change microarray
hsa-miR-106b	0.54	1.09
hsa-miR-107	0.70	0.68
hsa-miR-1280	1.15	3.01
hsa-miR-151-3p	1.02	2.05
hsa-miR-17*	0.59	0.60
hsa-miR-18a	0.40	0.42
hsa-miR-199a-5p	1.08	2.77
hsa-miR-20a	0.41	0.58
hsa-miR-20b	0.48	0.88
hsa-miR-30a	0.78	1.14
hsa-miR-362-3p	0.98	2.07
hsa-miR-550*	0.65	1.02
hsa-miR-664	0.65	0.83

correlation	0.93	

**Figure 2:**
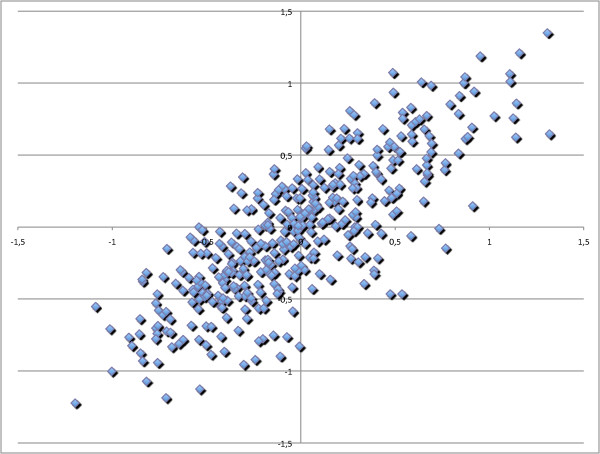
**Comparison of melanoma test and validation set**. Comparison of the miRNA expression in blood cells of melanoma patients in the test set and in the validation set. The logarithm of fold quotients of miRNAs was determined both for the 24 melanoma blood samples used as test set (x-axis) and for the 11 melanoma blood samples used as validation set (y-axis). The correlation of both fold quotients is 0.81.

We further compared the fold quotients in both the 24 melanoma blood samples that were used as test set and the 11 melanoma blood samples that were used as validation set. To reduce the noise, we only considered miRNAs with a median intensity level of at least 50 in any of the two sets. In this step we used a less stringent threshold of 50 to obtain more data points and little background noise has a less stringent influence on the correlation calculations. We computed for each melanoma set (i.e. test set and validation set) the fold quotient versus the controls and determined the correlation. The scatter-plot in Figure 2 presents the logarithm of fold quotients of the test set on the x-axis and of the validation set on the y-axis. The correlation of fold quotients between both melanoma test and validation set was as high as 0.81. These results demonstrate the reproducibility of the miRNA profiling in blood cells of melanoma patients.

**Figure 3:**
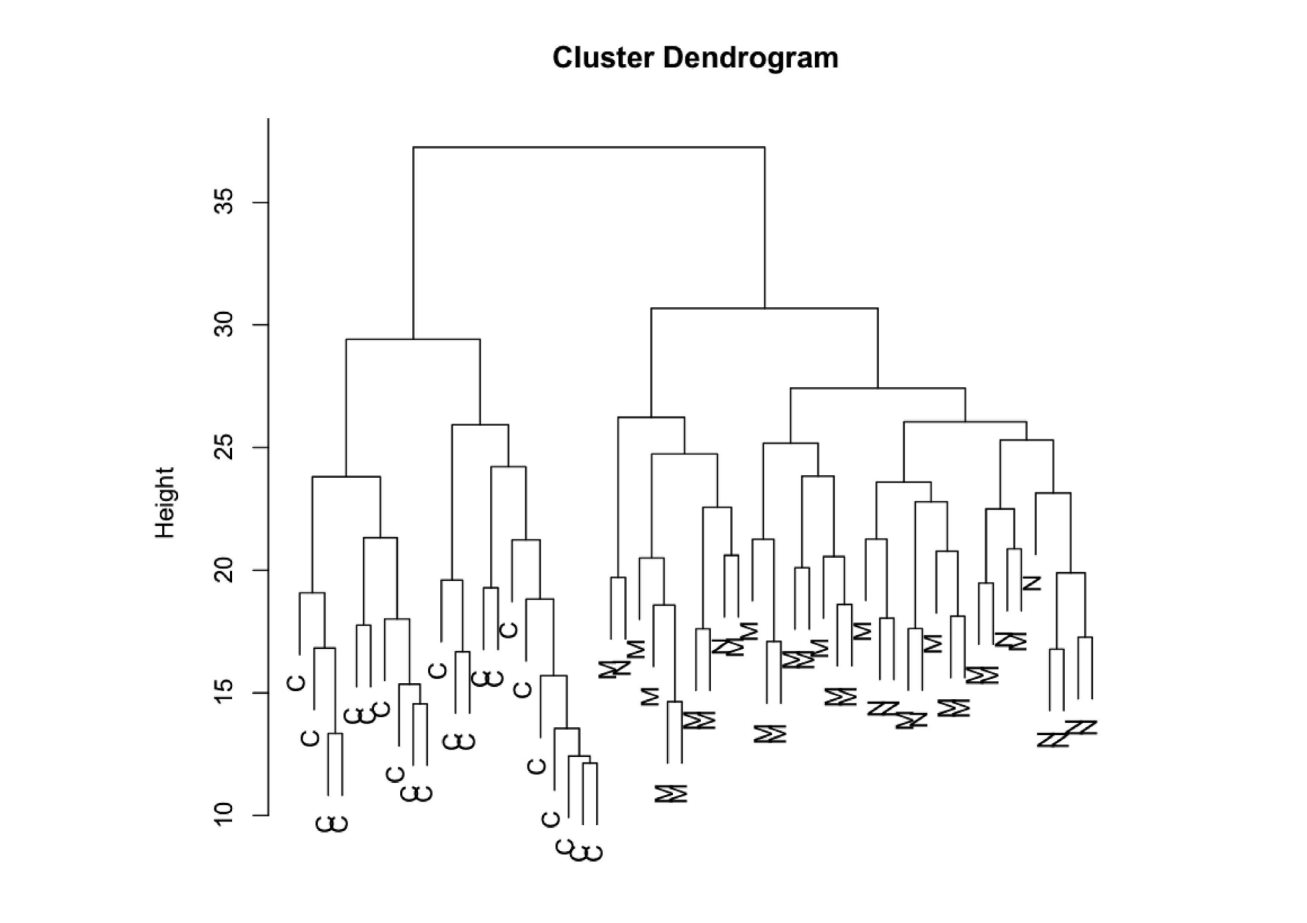
**Cluster analysis of all analyzed blood samples**. Cluster dendrogram of blood samples from healthy control subjects and from melanoma patients of the test and validation set. Cluster analysis was done for the 50 miRNAs with the highest data variance among all tested blood samples, i.e. samples of healthy controls (C), samples of the melanoma test set (M), and samples of the melanoma validation set (N). The healthy control subjects and the melanoma patients can be clearly differentiated.

We also analyzed the miRNA expression profiles of the melanoma test set, the melanoma validation set and the set of healthy controls by hierarchical clustering. Since many miRNAs contributed mostly noise to the clustering, we used only the 50 miRNAs with the highest data variance for clustering. As shown in the dendrogram in Figure 3, control samples and melanoma sample fall in two different major clusters. Melanoma samples of the test set and melanoma samples of the validation set are mixed within the same major cluster. Splitting the dendrogram in two groups and computing a contingency table we found that all control samples belong to one cluster and all melanoma samples belong to the other cluster. We obtained a p-value of approx. 3 * 10^-16 ^for this clustering using two-tailed Fisher's Exact test.

**Figure 4:**
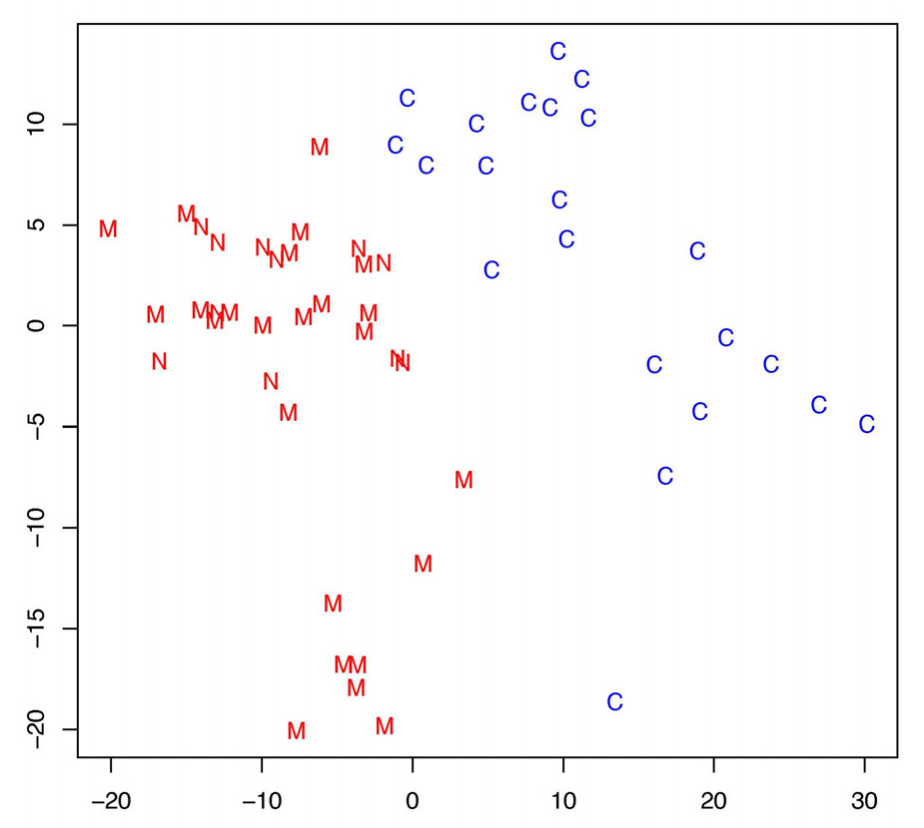
**Principal Component Analysis of all tested blood samples**. Principal Component Analysis of blood samples from healthy control subjects and from melanoma patients of the test and validation set. The figure shows the first (x-axis) versus the second (y-axis) principal component. Samples of healthy individuals are indicated by C, melanoma test samples by M, and melanoma validation samples by N. The healthy control subjects and the melanoma patients can be clearly differentiated.

To provide a low-dimensional visualization of the high-dimensional data we carried out a principal component analysis. Investigating the eigenvalues of the first principal components, we found that the first component contained the highest overall data variance while the first and second principal component combined contributed to approximately half of the overall variance. A plot of the first versus the second principal component is shown in Figure 4. The principal component analysis largely confirmed the results of the hierarchical clustering. Again, the control samples could clearly be differentiated from the melanoma samples.

**Figure 5:**
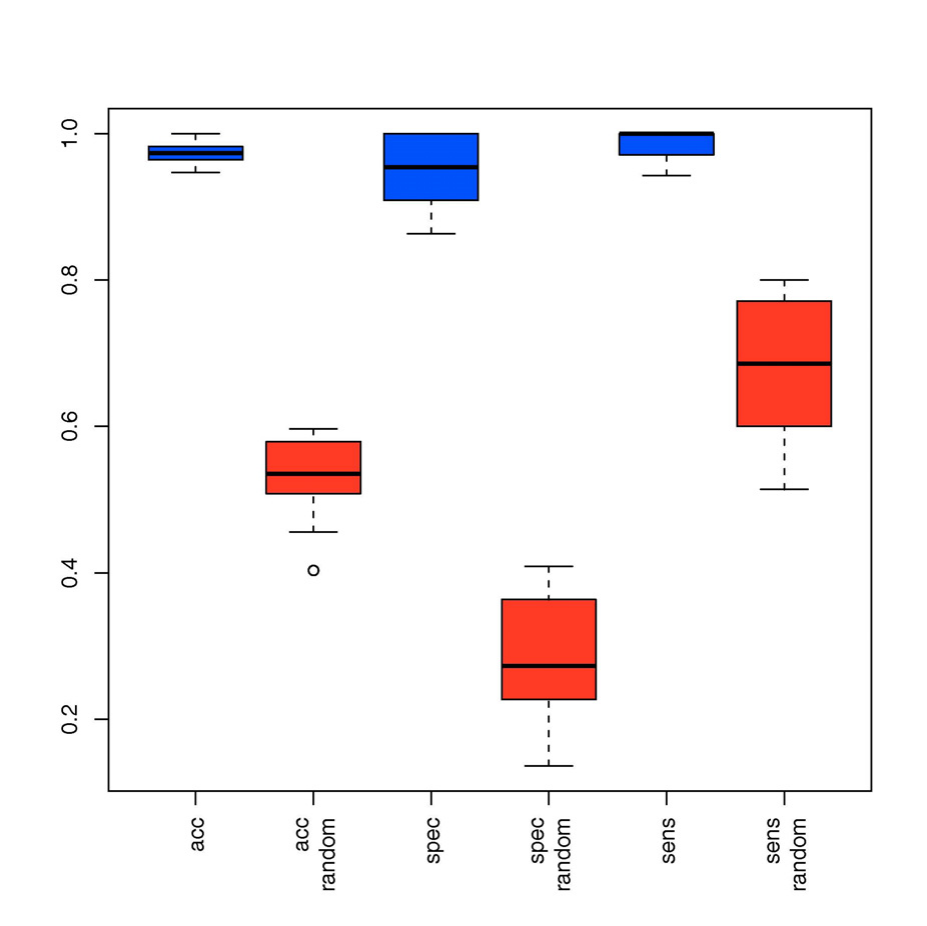
**Classification results**. Accuracy, specificity and sensitivity by which melanoma patients are identified based on miRNA profiling of blood. Blue boxes show the classification accuracy, specificity and sensitivity as determined by repeated cross-validation for the subset of 16 miRNAs. Red boxes show the respective accuracy, specificity and sensitivity for permutation test.

To confirm the results of the unsupervised cluster analysis, we employed a supervised statistical learning approach. We carried out SVM classification together with a feature subset selection relying on t-test p-values. In detail, we applied radial basis function SVMs that have been evaluated using 10-fold CV. The CV runs have been repeated 100 times to estimate the classification variance. To test for data overfitting, we carried out 100 permutation tests, i.e., we applied the same statistical approach to a data set with randomly assigned class labels for melanoma and control samples. The best classification accuracy has been obtained by using a subset that consists of 16 miRNAs including hsa-miR-186, hsa-let-7d*, hsa-miR-18a*, hsa-miR-145, hsa-miR-99a, hsa-miR-664, hsa-miR-501-5p, hsa-miR-378*, hsa-miR-29c*, hsa-miR-1280, hsa-miR-365, hsa-miR-1249, hsa-miR-328, hsa-miR-422a, hsa-miR-30 d, and hsa-miR-17*. By using these 16 miRNAs, we separated melanoma from healthy controls with a high accuracy, specificity and sensitivity of 97.4%, 95.0% and 98.9%, respectively. The results of all 100 CV runs and 100 permutation tests are provided as box-plots in Figure 5. For classification purposes the above mentioned 16 miRNAs do not have to meet the critera of an at least 2-fold deregulation and a combined median >100 in either all melanoma or all normal samples. Therefore, these 16 miRNAs are not necessarily a subset of the 51 differentially expressed miRNAs that were listed in Table 1.

**Figure 6:**
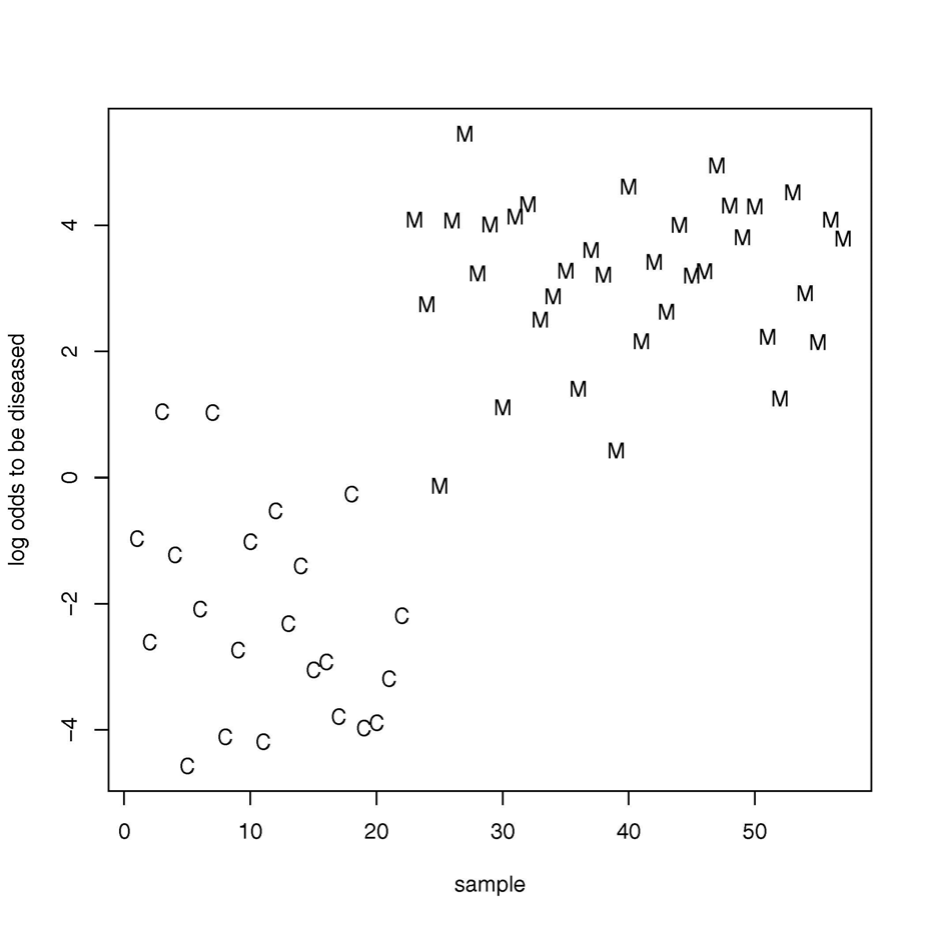
**Classification example**. Example for the classification of melanoma patients and healthy individuals based on the miRNA expression profiling of blood cells for the 16 miRNAs as detected by the subset selection. The logarithm of the quotient of the probability to be a melanoma patient and the probability to be a healthy individual is given on the y-axis for each control (C) and each melanoma (M). If this quotient is greater than one e.g. the logarithm is greater zero the sample is more likely to be a melanoma sample than a control sample.

An example of a classification result is shown in Figure 6. In detail, we determined the probability of being a melanoma patient or a healthy control based on the miRNA expression profiles of blood cells. The probability is calculated as the logarithm of the quotient of the probabilities to be diseased and the probability to be healthy. If the quotient of the probability is greater than one, e.g. the logarithm is greater zero, the sample is more likely to be a melanoma sample than a control sample. As shown in Figure 6, the majority of melanoma samples have logarithmized quotients of greater 0 while the majority of control samples have logarithmized quotients of below 0. These results further demonstrate that miRNA expression profiling of blood cells can separate melanoma patients from healthy individuals with high sensitivity and specificity.

## Discussion

One of the major challenges towards an improved melanoma treatment is the identification of appropriate markers for a most early detection of the primary lesion. For patients with stage I melanoma the overall 5-year survival rate exceeds 90% but can fall below 10% for stage III or IV melanoma. It is especially important to detect melanoma before metastasis that occurs early during melanoma progression. Equally important are the prognosis of the patients' outcome and the prediction of the response to treatment. Since melanoma is a very heterogeneous disease, complex biomarker profiles appear to be best suited for the task of early tumor detection and the monitoring of high-risk patients.

In our study we investigated the miRNA expression of almost all currently known human miRNAs and miRNA star sequences in peripheral blood cells of melanoma patients. The majority (88.57%) of the melanoma patients had a melanoma in clinical stage 0, IA, IB, IIA, or IIB. Comparing miRNA expression profiles in blood cells of melanoma patients and in blood cells of healthy donors, we detected 51 differentially expressed miRNAs. A total of 30 miRNAs was upregulated in blood cells of melanoma patients, whereas 21 miRNAs were downregulated. We used highly stringent selection criteria for the identification of deregulated miRNAs to reduce the false discovery rates, a problem that has recently been addressed by McCarthy et al. [32].

We compared our results with the data deposited in the Human miRNA and Disease Database (HMDD, http://202.38.126.151/hmdd/mirna/md/[33]). Most notably, the minority of the 51 deregulated miRNAs is annotated as cancer related miRNA in the HMDD. For example, hsa-miR-216a, the miRNA with second best AUC value has been described to be downregulated in lung neoplasm and is likewise more than 2-fold downregulated in melanoma in our study [34]. The miRNA hsa-miR-186, the upregulated miRNA with highest AUC has been described to be upregulated in pancreatic cancer [35]. However, most studies published so far differ significantly from our study in two factors: First, the majority of studies describes the miRNA expression analysis in cancer tissues, and second, most studies use less complete miRNA sets. Thus, it is evident that we also identified miRNAs as significantly deregulated in blood cells of melanoma patients that are not yet reported as deregulated in cancer or any non-cancer disease. One example is miR-1280 that is upregulated 2.5-fold in our studies but not deposited in the HMDD. Out of the 51 significantly deregulated miRNAs the minority of these miRNAs has been recorded in the HMDD as deregulated in any human cancer or non-cancer disease. In a study on genomic alterations that were related to miRNA genes, Zhang and colleagues reported 196 miRNA genes with copy number gains and 235 miRNA genes with copy number losses in melanoma [36]. They also showed a copy number alteration for the four miRNAs hsa-miR-214, hsa-miR-106b, hsa-miR-18a, and hsa-miR-20a, all of which were deregulated in melanoma blood cells as shown in our study. In detail, we found a downregulation of miRNAs hsa-miR-18a and hsa-miR-20a in blood cells of melanoma patients. Zhang et al. showed copy number loss for the corresponding miRNA genes. However, while we also found a downregulation of the miRNAs hsa-miR-214 and hsa-miR-106b in melanoma blood cells, Zhang et al. reported copy number gains for the corresponding miRNA genes. Without knowing the nature of the blood cells that give rise to the miRNA pattern, it is premature to speculate on a possible link between the miRNA pattern obtained from patients' blood and the pattern obtained from the tumor.

Out of the 51 miRNAs that were deregulated in blood of melanoma patients four miRNAs, namely hsa-miR-99a, hsa-miR-365, hsa-miR-30a, and hsa-miR-146a, were deregulated in non-cancer skin diseases [37,38]. The miRNAs hsa-miR-99a and hsa-miR-365 are downregulated in Lupus vulgaris [38], but were upregulated in blood of melanoma patients. The miRNA hsa-miR-30a was upregulated both in Lupus vulgaris [38] and in blood of melanoma patients. Likewise miRNA hsa-miR-146a was upregulated both in blood cells of melanoma patients and in non-cancer patients e.g. eczema patients [37]. The latter miRNA was also overexpressed in psoriatic lesional skin of psoriasis patients compared to healthy skin [39,40]. The miRNA hsa-miR-146a that contributes to an abnormal activation of type I interferon pathway in human lupus [41] was also upregulated in blood cells of melanoma patients.

While these first results do not allow any conclusion on the disease specificity of any of the addressed miRNAs, future miRNA profiling of a larger number of different human diseases will contribute to the identification of miRNAs that play a crucial role in specific human diseases. Furthermore, the identification of miRNAs will contribute to the overall understanding of the molecular alterations underlying disease development including melanoma progression.

In addition, and in keeping with the focus of this study, miRNA expression profiling especially of human blood has the potential to serve as future tumor biomarker. Applying SVM with a feature subset selection method we used a set of 16 miRNAs to differentiate melanoma patients and healthy blood donors with high accuracy (97.4%). Recently, we used a subset of 24 miRNAs to discriminate between blood cells of patients with lung cancer and healthy controls with an accuracy of 95.4% [10]. Except for lung cancer, there are no other studies that determine and compare miRNA profiles in blood cells of cancer patients and of normal controls [10,42]. A recent study by Chen et al. compared miRNA profiles in blood cells and in serum but did not separate cancer patients from normal controls by miRNA profiling [42]. Based on our studies melanoma patients can be separated from lung cancer patients and Multiple Sclerosis patients with approximately 90% accuracy by the miRNA expression signature. Ultimately, the classification accuracy depends on the choice of the control group and the choice of the control group depends on the questions to be answered by the miRNA expression profile. For example, investigating the usefulness of miRNA signatures as prognostic, predictive, pharmacodynamic or early detection biomarkers requires different control groups.

In this study we provide first evidence for the potential of miRNA expression profiles to distinguish patients with melanoma from healthy control subjects based on the analysis of peripheral blood cells. Until now it is not known which type of blood cells is responsible for the differences in the miRNA expression pattern. It is conceivable that the cancer miRNA signatures arise as part of a cancer-associated immune response. However, any hypothesis about the origin of the miRNA signatures generated from blood awaits experimental confirmation. Identifying the blood component responsible for specific miRNA signatures will likely contribute not only to our understanding of the mechanism underlying the pattern, but also to an improved prediction and prognosis of a disease.

Finally, it remains to be proven which cancers or non-cancer diseases also show a specific miRNA expression pattern that might be used to tell these diseases apart form controls and possibly apart from each other.

## Conclusions

Using a subset of 16 significant deregulated miRNAs, we distinguished melanoma patients from healthy individuals with an accuracy of 97.4%. The high specificity and sensitivity of the miRNA signatures generated for blood cells of melanoma patients underlines the potential of this approach for future diagnostic applications. Blood based miRNA signatures may be ideally suited as prognostic, predictive, or pharmacodynamic biomarkers not only for melanoma but also for other tumors.

## Abbreviations

AUC: area under the receiver operator characteristics curve; CV: cross validation; FP: false positive; FN: false negative; GRTA: Geniom Realtime Analyzer; HMDD: Human miRNA and Disease Database; limma: linear model with p-values computed by an empirical bayes approach; miRNA: microRNA; MPEA: microfluidic-based enzymatic on-chip labeling of miRNAs; PCA: principal componenet analysis; ROC: receiver operator characteristics curve; SVM: Support Vector Machines; TP: true positive; TN: true negative; WMW: Wilcoxon Mann-Whitney test

## Competing interests

AK: salary (febit), patent (febit); AB salary (febit), patent (febit); MS salary (febit); FW salary (febit); AW salary (febit);

## Authors' contributions

PL contributed to study design and developed the protocol for miRNA extraction, extracted the miRNAs from blood cells, and contributed in paper writing. AK contributed to study design, carried out the statistical analyses, initiated the study and contributed in paper writing. AB performed the miRNA screening. JR, KR, and SUJ collected the blood samples and contributed in paper writing. HPL contributed in the bio-statistical analysis. EM contributed in study design and development of miRNA extraction, supervised the study and contributed in paper writing. All authors read and approved the final manuscript.

## Pre-publication history

The pre-publication history for this paper can be accessed here:

http://www.biomedcentral.com/1471-2407/10/262/prepub

## Supplementary Material

Additional file 1 Additional Table S1: Detailed information on all blood donors, i.e. melanoma patients and healthy individuals. Click here for file
